# Some Remarks on Mentally Deficient Children

**Published:** 1909-05-08

**Authors:** Alfred Greenwood

**Affiliations:** Medical Officer of Health, Blackburn; Medical Officer Blackburn Education Authority, etc.


### SPECIAL ARTICLES.

### SOME REMARKS ON MENTALLY DEFICIENT CHILDREN.

By ALFRED GREENWOOD, M.D., B.Sc., D.P.H.; Medical Officer of Health, Blackburn; Medical Officer Blackburn Education Authority, etc.

A young child has aptly been compared to a plant during the processes of growth and development, and it is common knowledge that he, or she, may be affected for weal or woe by good or evil factors which may play important parts during those processes.

The various stages of development from infancy onwards in a normal child are well known. The child's intellect does not grow so quickly as the muscles and power of movement. At the same time the first signs of intellect show very early in babyhood. At first light can be distinguished from darkness, then colour can be appreciated; for instance, red or yellow is chosen in preference to black. Noises attract the baby's attention, and then, later, he can tell the parents' voices from those of strangers. The baby then develops the sense of distance, and soon finds out those things which he can lay hold of within reach and those which he cannot. Form and other properties of objects come later. Then the child begins to understand the meaning of certain words, and by and by tries to imitate these sounds. A fine new field of instruction is then opened up for the child, and the faculty of spontaneous speech is developed.

Thus during the bodily development there is also an increasing development of the brain in a normal child. A child may enter this world deprived of certain centres, or may exhibit signs of arrested development of various centres—*i.e.* all the faculties may not be equally developed. In very marked cases falling in this group the result is idiocy or imbecility; such children are easy to detect. There is, however, another group of children who occupy various intermediate positions between those who are normal and those who are so far removed from the normal state of mental capacity and development as to be incapable of receiving benefit from special educational measures. According as these children are at the upper or lower end of their own class they present varying degrees of difficulty, from the points of view of diagnosis, treatment, or administration. For example, it is often difficult to decide whether or not certain children are or are not suitable for admission to a special school.

In this paper I shall not refer to physically defective or epileptic children. As is well known, power has been given, in the Defective and Epileptic Children Act of 1899, to a school authority to make such arrangements as are thought fit for ascertaining what children in their district, not being imbecile and not being merely dull or backward, are defective. That is to say, what children, by reason of mental or physical defect, are incapable of receiving proper benefit from the instruction in the ordinary public elementary schools, but are not incapable by reason of such defect of receiving benefit from instruction in special classes or schools. This Act is therefore a permissive one.

It will be seen from this that, according to the standard adopted in various districts in selecting children for admission to special schools so might the result vary. For example, on the one hand a certain special school might contain a preponderance of children nearer the imbecile state than the dull or backward state. On the other hand, a special school might contain a much larger proportion of children nearer the dull or backward state than the imbecile state. Yet both these schools might contain children in compliance with the Act of Parliament named above. But the results, as shown by educational progress, by the after-history, and by the numbers transferred to the ordinary elementary schools, would be far greater in the second school mentioned than the first one.

The possibility of such an occurrence would seem to indicate the need for an adoption of a uniform standard of mental capacity. This is, however, by no means easy to fix, and the only practical solution of this difficulty, it seems to me at present, is to proceed along certain lines made as definite as it is possible to make them when dealing with the vagaries of the highly organised and complex nervous system. It would also appear desirable that the improvable element should preponderate in these special schools as far as possible. Unless great care is taken, unsuitable children may gain admission.

There is no doubt that heredity plays an important part in the transmission of various peculiarities from parent to offspring. The province of heredity has become so large as to include not only diseases (marked instances of which are rheumatism and gout) but also instincts, sentiments, passions, memory, imaginations, and all manner of peculiarities of form and function. Everyone knows the likeness between many children and their parents in figure, form, gait, manner, and voice. And those who have experience of special schools have often been impressed by the marked lack of mental development in the parents of many of the scholars. Of course, the law of inheritance does not always hold good, and there are many instances of mental deficiency in children in which the cause is obscure.

Perhaps here it may be of interest if I point out a few details which assist members of the medical and teaching professions in selecting such suitable cases. Much useful information can be gained from the general aspect and nutrition of the child, and from the features, gait, and facial expression. The report of the child's teacher at the ordinary elementary school is a valuable help. A reliable family history respecting each child is also a great assistance. Dr. Fletcher Beach has classified the characteristics to be noted in cases of feeble-minded children as physical, mental, and moral. The form or shape of the head is the first physical characteristic mentioned. This may be too small, measuring only 17 or 18 inches in circumference, instead of the normal measurement, which is about 20 inches. The head in these cases is conical in shape, and the back of the head flattened. The forehead is narrow from side to side, and often recedes, but the features are usually shapely, the eyes large, and the nose aquiline. Many of this class are lively, restless, imitative, and fond of music, but are unable to learn much because they lack power of attention. On the other hand, some children have heads which are too large, and are round, with the largest circumference at the temple. Many of these do not improve much, but are generally good tempered and affectionate. Other children have one side of the head distinctly flattened.

It is important, also, to note the condition of the hard palate, which in the majority of feeble-minded children presents marked differences from that of other children. The deformities noted in feeble-minded children consist mainly in a high, narrow, and V-shaped form. Again, the ears in feeble-minded children may be too long, asymmetrical, or deprived of the lower portion or lobule. The tongue, teeth, and lips may be too large and there may be asymmetry of the limbs. The skin may contain too much fat or it may be dry and rough. The hair is also frequently dry and brittle. The latter condition is probably an indication of the lower vitality which is more prevalent amongst these children than amongst other children. The power of attention varies in many children presented for examination. If it is impossible to fix a child's attention one may generally conclude that satisfactory results are unlikely to be gained at a special school.

The speech is very often defective, and the senses of smell, taste, touch, and hearing are often imperfect. A considerable number of backward children suffer from post-nasal adenoids, enlarged tonsils, and inflammation of the middle ear. These conditions frequently lead to deafness and backwardness, but they are generally capable of remedy, and therefore should be attended to whenever possible.

I will now deal briefly with those children who gain admission to one of these special schools. Owing to the smaller classes, the specially trained and highly qualified teachers necessary, and the greater cost of administration generally, each boy or girl in a special school costs more than those in the ordinary public elementary schools. Naturally the ratepayer will say, " What return will there be for this expenditure? " In answering this question it should be remembered that there can be no immediate return, and that the results appear tardily after long and patient endeavour. Having overcome the objections of the parents (and this is by no means easy, especially in a town where the first special school is commencing), the child begins to receive special tuition. It should be stated here that the scholars of special schools are " not idiots, nor imbeciles, but through some mental defect they are incapable of receiving proper benefit in an ordinary public elementary school." Such terms as " mad school " or " silly school " are, therefore, entirely out of place in reference to these institutions. The kind of work carried out in these schools will be far more appreciated by a few personal visits than by any description. Children who show a special aptitude for a certain subject, as manual work or domestic work, are encouraged to develop their faculties in those particular directions. As many of these special schools are buildings which have been erected in comparatively recent years, the scholars spend their school lives under excellent hygienic conditions. The well-equipped bathrooms at the Blackburn special school have worked wonders amongst the children. Over 40 boys and girls are bathed weekly, and it is worthy of record that there is not one dirty head in the school. This result has not been achieved, however, without much patient work on the part of the teachers. At first many of the children were afraid of the bath, and had to be coaxed to watch the performance before submitting to the bath themselves. Now it is a punishment for the bath to be withheld. Also, the children are much better able to dress themselves than when they attended this school first. Children who were formerly brought to school have been encouraged to come alone as soon as they knew the route and when it was considered safe. It is important that a spirit of self-reliance and independence should be fostered in the scholars whenever possible.

There is no doubt that many children attending special schools are living under bad home conditions, which militate against the benefit received at school. This is very unfortunate, and I am afraid it can only be remedied by removing these children from their homes altogether. It is, however, not desirable that parental responsibility should be diminished unduly, and parents who do not do their duty when they are able should be punished without any considerations of false sentiment.

Referring now to the results gained from special schools, it has been proved beyond doubt in London and elsewhere that although there have been some failures, the general result has been satisfactory as showing that these children repay the money expended upon them during school life, for without it they would have been unable to help themselves on leaving school. For example, after such training many children have found employment; the girls as general servants, laundresses, dressmakers, etc., and the boys as errand-boys, labourers, vanboys, workshop employees, etc. It is at this stage that the teachers and others interested can do so much good in forming after-care associations, whose objects should be to find employment for children leaving special schools, and exercise a philanthropic supervision over them subsequently.

In addition to these results many children who have received tuition in special schools for varying periods have improved so much that they have been transferred to the public elementary schools, where they have continued to improve. In this connection it should be mentioned that the teachers in the public elementary schools to whom such children are transferred should take a real interest in them, otherwise the efforts of the previous special teaching may be useless.

From the above remarks it will be quite obvious that children are often presented for medical examination with a view to admission to a special school whom it would be useless to send there owing to incapacity for improvement, mentally and morally. It is very important that these children should be taken in hand, both for their own sakes and for the sake of the community. The question now follows naturally, What should be done with the failures? Although this is a difficult question, it will have to be faced boldly. In brief, my own view is that the remedy can only be found in the direction of compulsory, permanent detention in some institution where the lives of these "failures" may be made as comfortable and harmless as possible, and especially where there can be no opportunity for marriage and propagation of this type. This would seem to be especially desirable in the group of cases known as moral imbeciles, who are often very clever, baffle their guardians, and develop into dangerous criminals.

Respecting the subsequent marriage of the mentally deficient, there appear to be various opinions. Dr. Shuttleworth states in his book on "Mentally Deficient Children" that of nearly a thousand discharged patients who had passed under observation after institution training, only two were known to have married, and that his experience lent no support to the view which is urged as an objection to educating mentally deficient children and fitting them for work in the world, that they would be thereby encouraged to marry, and in consequence there would be a risk of multiplying mental defect in the progeny. It appears to me, however, that the danger of marriage is a real one, and if one remembers the number of mentally deficient children where heredity has played a part, one must be impressed with the desirability of preventing such a continuance of this type if possible.

Miss Dendy, whose excellent work in Manchester is so well known, has made several suggestions on this point—namely, that the system of teaching in special schools should be made almost entirely a training in manual work, manners, and morals, and that there should be in connection with every special school a residential special school or colony. She recommends that every child should be dealt with by the doctor, not on the ground of its attainments, but on the ground of its mental condition, and that for every child whom the doctor cannot certify, at the age of 13 or 14, that it is a normal child, a place in a residential institution should also be found where it should remain, on a renewable certificate, for life. Miss Dendy states that the plan would not be any extra expense to the community, but that, on the contrary, it would be a saving of expense, as it would cost much less to keep these people this way than in workhouses and gaols. She also suggests that a trade school might very well be the intermediate step between the special school and the colony, as this would give a further opportunity to those children whom the doctor might consider normal, except in the power of acquiring learning.

